# Impact of AYUSH interventions on COVID-19: a protocol for a living systematic review and meta-analysis

**DOI:** 10.12688/f1000research.55109.1

**Published:** 2021-07-28

**Authors:** Anup Thakar, Kalpesh Panara, Mandip Goyal, Ritu Kumari, Kim Sungchol

**Affiliations:** 1 Institute of Teaching and Research in Ayurveda, Jamnagar, Gujarat, 361008, India; 2 World Health Organization, Regional Office for the South East Asia, Indraprastha Estate, Mahatma Gandhi Marg, New Delhi, 110002, India

**Keywords:** Ayurvedic medicine, AYUSH, Complementary therapies, COVID-19, Systematic review and meta-analysis

## Abstract

**Background: **The coronavirus disease 2019 (COVID-19) pandemic has created a great burden on governments and the medical fraternity globally. Many clinical studies from the Indian system of Traditional Medicines [Ayurveda, Yoga and Naturopathy, Unani, Siddha, and Homoeopathy (AYUSH)] have been carried out to find appropriate solutions. Through a living systematic review and meta-analysis, this study aims to determine the effectiveness of the Traditional System of Indian Medicine (AYUSH system) in lowering the incidence, duration, and severity of COVID-19.

**Methods:** We will search the following databases: Pubmed; the Cochrane central register of controlled trials (CENTRAL); the Clinical Trials Registry - India (CTRI); Digital Helpline for Ayurveda Research Articles (DHARA): AYUSH research portal; and World Health Organization (WHO) COVID-19 database. Clinical improvement, WHO ordinal scale, viral clearance, incidences of COVID-19 infection, and mortality will be considered as primary outcomes. Secondary outcomes will be use of O2 therapy or mechanical ventilator, admission to high dependency unit or emergency unit, duration of hospitalization, the time to symptom resolution, and adverse events. Two authors will independently search the articles, extract the data and disagreements will be resolved by the involvement of a third reviewer. Data will be synthesized, and the risk of bias will be assessed with RevMan 5.4 tool. Certainty of evidence will be assessed through the GRADE (Grading of Recommendations, Assessment, Development and Evaluations) tool. The review will be updated bi-monthly with two updates.

**Conclusion:** This living systematic review will be the first to address AYUSH interventions in COVID-19, synthesizing the full spectrum of Indian Traditional System of Medicine against COVID-19. It will facilitate professionals, guideline developers, and authorities with up to date synthesis on interventions periodically to make health-care decisions on AYUSH therapies in the management of COVID-19.

## Introduction

The pandemic of severe acute respiratory syndrome coronavirus 2 (SARS-CoV-2), causing coronavirus disease 2019 (COVID-19) has expanded over the globe, affecting most countries in the world, and led to significant morbidity and mortality. Mutation in SARS-CoV-2 within its transmissible form has been detected in some continents leading to increased public health distress. ^1^ Scientists throughout the world are rigorously engaged in the development of effective vaccines and therapeutics for the prevention and cure of this novel coronavirus. Statistics indicate that despite efforts undertaken by various health care professions and authorities, cases are still on the rise. ^2^

People are turning to alternative treatments for prevention or cure because there is no promising medication accessible. Research on COVID-19 from Alternative and Complementary Medicines are being carried out in many countries. ^3^ Countries including India, China, and South Korea, have issued guidelines on traditional medicines for the prevention and management of COVID-19. ^4^ Several initiatives have been launched to support ongoing research in the Traditional, Integrative, Complementary and Alternative Medicine (TICAM) to utilize available traditional knowledge in an integrated manner. ^5^^,^^6^ Ayurveda, Yoga, Naturopathy, Unani, Siddha, and Homeopathy (abbreviated as AYUSH) ^7^ are five alternative and complementary therapies prevalent in India that are widely used in COVID-19 management. At inception of the pandemic, ministry of AYUSH (regulatory body of Indian system of medicine) issued advice based on an advisory panel of AYUSH experts and primitive evidence that recommended the use of some herbs and measures to enhance immunity. ^8^ In this advice, traditional herbs and measures, which have already been in use for decades for various ailments like fever, cough, and respiratory distress, and as an non-specific immunity enhancer, possessing anti-viral, anti-bacterial and anti-microbial properties, were recommended. ^9^ Among recommended formulations, some have undergone scientific investigations, such as Ayush 64, Chyawanprash, Guduchi Ghanavati, Arsenica Album, Kabasur Kudineer, Nilavembu Kudineer, for their possible preventive or therapeutic impact. ^10^ Some trials on AYUSH interventions are already completed and published ^11^^–^^13^ or in press. Findings of such studies need to be appraised and summarized carefully through syntheses of evidence to determine the strength of the evidence. Further, it is time for AYUSH health policy makers to examine and revise the guidelines recommended for COVID-19 using an evidence-based tactic, involving the best research existing till date. This study aims to assess the effectiveness of the Traditional System of Indian Medicine (AYUSH systems of Medicine) on reducing the incidence, duration, and severity of COVID-19 through systematic review and meta-analysis. Traditional systematic reviews provide an overview of the relevant evidence at a specific time only, whereas living systematic reviews address this limitation through periodical updates. A living systematic review provides a thorough and current appraisal of the evidence that may help to develop and update recommendations and clinical guidelines time to time.

## Protocol

This protocol has been registered in PROSPERO ( CRD42021244831) prospectively.

## Eligibility criteria

All clinical trials, observational (analytical) researches on any interventions of the AYUSH systems published in English language only, regardless of publication status, will be included in our study.

### Participants

Person with risk of COVID-19 exposure or with suspected, probable, or confirmed COVID-19 will be included independently of the severity of their symptoms, gender, age, or ethnicity.

### Interventions

Any type of intervention or exposure from any of the AYUSH system of medicine aimed at prophylaxis or treatment either stand-alone or add-on to the comparator (standard of care or placebo or no treatment control) will be included in our study. There will be no restriction regarding dose, dosage form, duration of treatment or number of medicines used.

### Outcome measures

Studies done on AYUSH interventions intended for both prophylaxis and therapeutic purposes. Therefore, we will divide our outcome measures in two categories.

• Primary outcomes for therapeutic studies will be clinical improvement, ordinal scale for disease severity, mortality and viral clearance; and for prophylaxis studies will be incidence of COVID-19 infection and mortality.
• Secondary outcomes for therapeutic studies will be use of O _2_ therapy, use of ventilator, admission to high dependency unit or emergency unit, duration of hospitalization, the time to symptom resolution, and adverse events; and for prophylaxis studies will be symptomatic SARS-CoV-2 infection, disease severity and adverse events.

## Information sources

We will search the following databases: Pubmed; the Cochrane Central Register of Controlled Trials ( CENTRAL); WHO COVID-19 database; the Central Trial registry - India ( CTRI); Digital Helpline for Ayurveda Research Articles ( DHARA); and AYUSH research portal. These databases will be searched from 1st December 2019 till the current time. We will restrict our studies to the studies published in English only without any publication restrictions. Hand searches will be conducted on the reference lists of eligible primary studies. Preprints ( SSRN, OSF, medRxiv), grey literature ( ayurCASERxiv) and unpublished literature will be searched.

### Search strategy

Search terms will be as follows: “COVID - 2019” OR “SARS-CoV-2” OR “NCP” OR “Corona Virus Disease-19” OR “COVID-19” AND “Indian Traditional Medicine” OR “AYUSH” OR “Ayurveda” OR “Yoga Naturopathy” OR “Unani” OR “Siddha” OR “Homeopathy”. A combination of medical subject headings [MeSH] terms and other text words will be used. Full search strategies with preliminary results are summarized in the *Extended data*. ^14^

## Data collection

Endnote X9 software will be used to manage the citations searched from the various databases. Two reviewers will independently screen all titles and abstracts. Of those articles selected by at least one of the reviewers, each of them will independently apply an inclusion and exclusion criteria checklist to decide if the study meets our selection criteria.

**Figure 1.**
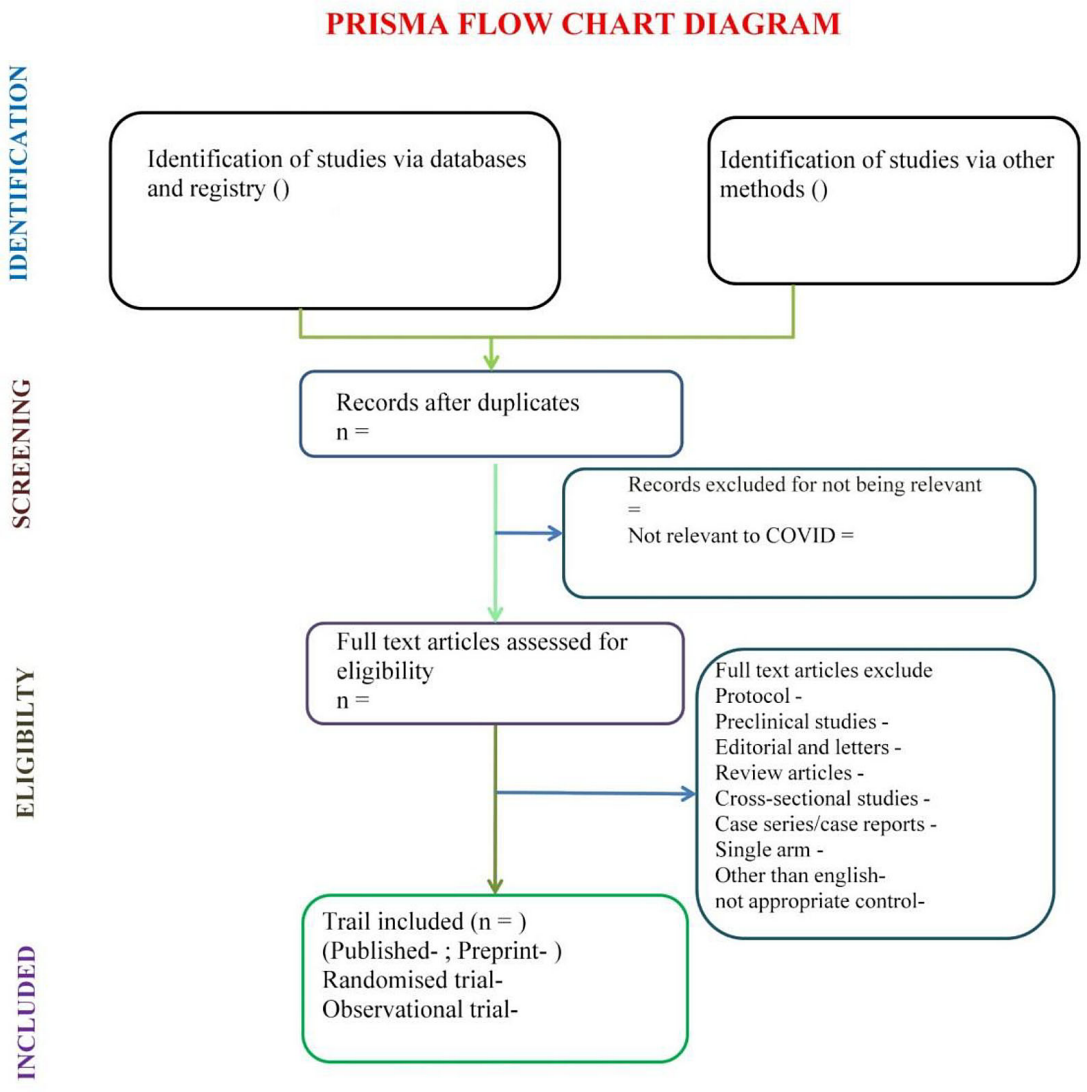
PRISMA flow chart for study selection process. Flow chart covers the plan of study selection process of living systematic review.

Articles identified via different databases, registry and other methods will be collected and processed through Endnote X9 software wherein duplicates and irrelevant articles will be removed. Remaining article will be reviewed full text. Protocol, pre-clinical, cross-sectional, case reports, case series, single arm or not having appropriate control will be excluded. Articles published in language other than English will also be excluded. Included studies will be categorized according to publication status and methodology and processed for systematic review. Study selection process is displayed graphically in the PRISMA-P flow diagram ( Figure 1). Disagreements will be resolved by discussion between the two reviewers, with a third person if consensus cannot be reached.

For the short listed articles two reviewers independently extract the data with reference to name of study, place of study, type of study, inclusion criteria, exclusion criteria, number of participants randomized, dose, frequency, route of administration and duration for each intervention, number of participants who received each intervention, comparators, baseline data, mean age, percent male, severity of illness (mild, moderate, severe, critically ill), co-morbidities outcomes, duration of interventions, number of participants, and methodological characteristics. A pilot-tested standardized data extraction form with detailed instructions has been developed ( *Extended data* ^14^). Any disagreement will be resolved by consensus or the involvement of the third assessor.

## Assessment of risk of bias

The risk of bias of the included studies will be done using the Cochrane Risk of Bias tool RoB 2.0, ^15^ which includes consideration of the following items: randomization process, deviation from intended intervention, missing outcome data, measurement of outcome, and selection of reported results. Non Randomized Studies of Interventions (NRSIs) will be ranked for risk of bias using Risk Of Bias In Non-randomized Studies - of Interventions ( ROBINS-I) tool ^16^ and domains are: bias due to confounding; bias in selection of participants; bias in classification of interventions; bias due to deviations from intended interventions; bias due to missing data; bias in measurement of the outcome; and bias in selection of the reported result. Across these domains we will rate the risk of bias of studies at i) low risk of bias, ii) some concerns, and iii) high risk of bias. When there is a low risk of bias across all domains, overall risk of bias will rank as low risk; when at least one domain bears some concerns, overall risk of bias will rank as some concerns; and studies will be ranked as high risk when at least one domain falls into the high risk category or multiple domains fall into the some concern category. Any differing views will be discussed with another team member.

## Effect measures

We will analyze our data in accordance with the Cochrane Handbook for Systematic reviews of Interventions. ^17^ We will use the Risk Ratio or Odds Ratio to compute relative impacts for outcomes with dichotomous data. For continuous outcomes, we will utilize mean difference and standard deviation (with 95% confidence intervals). If the unit of any of the measures isn't consistent throughout the studies, we will convert it to a standardized value for analysis.

## Dealing with missing data

Whenever we find insufficient or any missing data then the authors of the studies will be contacted for clarification, with one follow-up email. If we do not receive satisfactory answers then we will assume data to be missing at random and analyze only the available data (i.e. ignoring the missing data).

## Data synthesis

The characteristics and findings of the included studies will be presented in tables that summarize the study design, intervention, study participants, and outcomes. Meta-analysis will be displayed in forest plot. RevMan software 5.4 ^18^ will be used for various task of data analysis such as measurement of effects, assessment of heterogeneity, sub-group analysis, sensitivity analysis and for assessment of reporting bias.

## Assessment of heterogeneity

Testing for heterogeneity between the studies will be done by using Cochran’s Q test and by I ^2^ test statistics. Heterogeneity will also be assessed by visual assessment of forest chart.

## Subgroup analysis

The subgroup analyses will be carried out for age category (young, middle, old), disease severity (mild, moderate, severe) and dose of the interventions, if possible.

## Sensitivity analysis

Sensitivity analysis will be performed to test the robustness of findings that are not affected by the different decisions that could be made during the review process. Sensitivity analysis has been planned considering risk of bias.

## Assessment of reporting bias

For a specific direct comparison, funnel plot assessment for publication bias will be done when there will be ten or more than ten studies available. Any asymmetry of funnel plot will signify possible small research effects and thus will enable us to be aware about the small study bias.

## Confidence in cumulative evidence

Grading of Recommendations Assessment, Development and Evaluation (GRADE) methodology will be used for the assessment of evidence level of the results. Factors that are considered to analyze the quality of the evidence include research limitations, effect consistency, imprecision, indirectness, and publication bias. The evidence quality will be categorized as high, medium, low and very low.

## Updates of living systematic review

We plan to run searches for new studies every month. This will also include screening abstracts of the recently retrieved reports. The monthly interval for screening was preferred as we expect a rise in appropriate publications. The review itself will be updated every two months, providing that a sufficient quantity of new records will be acknowledged for inclusion. Living review will be ceased after two updates, then its necessity will be reanalyzed. It may be only continued further if additional budget provided by funder.

This is living systematic review, so, there is no need for ethical approval. There is no direct involvement of human or animal participants. This review will be disseminated in a peer reviewed journal.

## Study status

Preliminary searches from databases and the study selection process have been completed, data are being analyzed and synthesized presently.

## Conclusion

This living systematic review will be first review addressing AYUSH interventions in COVID-19 in which the full spectrum of Indian Traditional System of Medicine against COVID-19 will be summarized. It will facilitate clinicians, guideline developers, and policymakers to take health care decisions on AYUSH interventions in COVID-19 management. The reliability and validity of the findings will mainly depend on the variability in Population, Intervention, Comparator, Outcome (PICO) of primary evidence included and methodological quality among them. We plan to include pre-prints due to the importance of the information and the fact that many studies will likely be first published in pre-print repositories. We can expect the possibility of publication bias as positive outcome studies are more likely to be published sooner than negative outcome studies; however, including pre-prints may reduce publication bias.

## Data availability

### Underlying data

No data is associated with this article.

### Extended data

Zenodo: Data set for AYUSH interventions for COVID-19- A Living Systematic Review and Meta-analysis, https://doi.org/10.5281/zenodo.5091828. ^14^

This project contains the following extended data:

- Data extraction tool
- Search strategy

### Reporting guidelines

Zenodo: PRISMA-P Checklist of protocol - AYUSH interventions for COVID-19 - A Living Systematic Review and Meta-analysis, https://doi.org/10.5281/zenodo.5109089. ^19^

Data are available under the terms of the Creative Commons Attribution 4.0 International license (CC-BY 4.0).
