# Supplementary material for: Impact of AYUSH interventions on COVID-19: a protocol for a living systematic review and meta-analysis
Source: F1000Res. 2022 Mar 8;10:674. Originally published 2021 Jul 28. [Version 2] doi: 10.12688/f1000research.55109.2 (PMC8822137; doi:10.12688/f1000research.55109.2)
Supplement: Peer review history

# Peer review: Reviewer response for version 2

**DOI:** 10.5256/f1000research.122040.r126656

**Published:** 2022-04-04

**Authors:** Ram C. Bajpai

**Affiliations:** 1 School of Medicine, Keele University, Keele, UK

Authors are planning to include all types of study designs (such as trials, cohort studies, and case-control studies) which is not recommended if you read the Cochrane Handbook carefully. A substantial methodological heterogeneity will make meta-analysis results useless and unreliable. Clinical trials and prospective observational can only be combined as their designs are similar. Authors must choose to do a systematic review of trials (may include prospective cohorts), or a systematic review of observational studies only (cohorts and other retrospective designs). High-quality evidence is solicited from any systematic review rather than low-quality evidence by colliding everything in an unexplained manner. Authors must think about it and make the right decision and modify the entire protocol here and in the PROSPERO.

 I couldn't see the end date for the literature search in the information sources. It has to be a final date by which all databases will be searched.

 The data synthesis section is too broad at the moment. Authors must write it according to the nature of outcomes variables (such as continuous and binary outcomes). They need to specify meta-analysis method by type of outcomes

Is the study design appropriate for the research question?

Yes

Is the rationale for, and objectives of, the study clearly described?

Yes

Are sufficient details of the methods provided to allow replication by others?

No

Are the datasets clearly presented in a useable and accessible format?

Yes

Reviewer Expertise:

Epidemiology, Medical Statistics, Meta-analysis

I confirm that I have read this submission and believe that I have an appropriate level of expertise to confirm that it is of an acceptable scientific standard, however I have significant reservations, as outlined above.

# Peer review: Reviewer response for version 2

**DOI:** 10.5256/f1000research.122040.r126658

**Published:** 2022-03-28

**Authors:** Rohit Sharma

**Affiliations:** 1 Department of Rasa Shastra and Bhaishajya Kalpana, Faculty of Ayurveda, Institute of Medical Sciences, Banaras Hindu University, Varanasi, Uttar Pradesh, India

The authors have addressed my comments and I am satisfied with the improvements.

Is the study design appropriate for the research question?

Yes

Is the rationale for, and objectives of, the study clearly described?

Yes

Are sufficient details of the methods provided to allow replication by others?

Yes

Are the datasets clearly presented in a useable and accessible format?

Yes

Reviewer Expertise:

Ayurveda, Herbal Medicine, Traditional Medicine, Natural Products, Complementary and Alternative Medicine

I confirm that I have read this submission and believe that I have an appropriate level of expertise to confirm that it is of an acceptable scientific standard.

# Peer review: Reviewer response for version 1

**DOI:** 10.5256/f1000research.58655.r122001

**Published:** 2022-02-07

**Authors:** Dr. Galib Ruknuddin

**Affiliations:** 1 Department of Ras Shastra and Bhaishajya Kalpana, All India Institute of Ayurveda, University of Delhi, New Delhi, Delhi, India

A rational topic for systematic review. Evaluating the efficacy of AYUSH interventions by analyzing the best available evidence is the need of the hour. The outcome may significantly contribute to the suffering population. However, the present protocol may consider a few of the below: 

  

1. Eligibility criteria for 'control' may be included.
2. Excluded studies may be mentioned in the eligibility criteria during the study design.
3. Detail planning on data synthesis methods is needed, focusing on how RCT and NRSI will be combined in meta-analysis and interpreted.
4. The author needs to clarify the selection of the model in data analysis and synthesis.

Is the study design appropriate for the research question?

Yes

Is the rationale for, and objectives of, the study clearly described?

Yes

Are sufficient details of the methods provided to allow replication by others?

Yes

Are the datasets clearly presented in a useable and accessible format?

Yes

Reviewer Expertise:

Ayurveda

I confirm that I have read this submission and believe that I have an appropriate level of expertise to confirm that it is of an acceptable scientific standard.

# Response

**Published:** 2022-03-02

**Authors:** Kalpesh Panara

**Affiliations:** Institute of Teaching and Research in Ayurveda, India

First of all, we are gratefully appreciating your invaluable suggestions in improving the quality of our research article. The responses to your observation and suggestion are as follows:

**Comment #1:** Eligibility criteria for ‘control’ may be included.

**Response: **As per the suggestion of the respected reviewer, eligibility criteria for the control has been added as:

*This review will include all the trials with the appropriate control as Standard of care or placebo or no treatment. Uncontrolled studies or AYUSH drugs as control will be excluded.*

**Comment #2:** Excluded studies may be mentioned in the eligibility criteria during the study design.

**Response: **We value the suggestion of the respected reviewer and added the type of study which will be excluded as:

*Protocol, pre-clinical, cross-sectional, case reports, case series, single-arm or not having appropriate control will be excluded. Articles published in a language other than English will also be excluded*

**Comment #3: **Detail planning on data synthesis methods is needed, focusing on how RCT and NRSI will be combined in meta-analysis and interpreted.

**Response: **We agree that RCTs and NRSIs are not combined routinely in meta-analysis except in exceptional circumstances. As limited RCTs on Ayush interventions are anticipated, which may restrict us from performing a meta-analysis, we opted to add NRSI and RCT for meta-analysis. It may lead to induce clinical heterogeneity in the pooled outcome measure. Further, assuming heterogeneity due to different designs, we have planned a random effect model to compute the summary effect. Moreover, we will perform sensitivity analysis considering the study design to know the impact of the inclusion of NRSI on pooled estimates. Further, if sufficient RCTs will be available in subsequent updates, we may exclude NRSIs from data syntheses.

 The intent of this reply is added and highlighted with green colour in the revised manuscript.

**Comment #4:** The author needs to clarify the selection of the model in data analysis and synthesis.

**Response: **We thank our reviewer for their observation that the selection of the model in data analysis and synthesis should be more explicit.

 Concerning the data analysis and synthesis, our submissions are as follows:

 As the anticipated nature of studies may be diverse, heterogeneity may be observed. Therefore, we have planned a random effect model to compute the summary effect. Additionally, Sensitivity analysis based on the nature of the study will be done to explain the heterogeneity, if sufficient number of study available.

 The intent of this reply has been added and highlighted in the manuscript for an easy review.

 All the additional phases have been highlighted in green in the revised manuscript for easy review.

 Finally, the authors are warm-heartedly admiring your invaluable comments/suggestions and believe that your kind contributions have undisputedly helped us to improve the quality of the research article.

**Yours sincerely**

**Authors**

# Peer review: Reviewer response for version 1

**DOI:** 10.5256/f1000research.58655.r99651

**Published:** 2021-11-24

**Authors:** Ram C. Bajpai

**Affiliations:** 1 School of Medicine, Keele University, Keele, UK

Authors must be more specific about the study designs eligible for this systematic review. For example, it is completely unclear whether a large case-series study will be eligible or not. So, kindly elaborate on your inclusion in more detail.

 Authors cannot ignore Google Scholar for searching eligible articles, as it is one of the biggest databases for grey literature and lots of Indian papers published in non-indexed journals.

 What do you mean by ‘till current time’ for searching different databases? There has to be an end date in the protocol.

 How was clinical improvement defined for this systematic review? Kindly explain in more detail.

 The statement, “Protocol, pre-clinical, cross-sectional, case reports, case series, single-arm or not having appropriate control will be excluded. Articles published in a language other than English will also be excluded.” Should be mentioned in the eligibility section.

 Authors must adhere to the PRISMA 2020 reporting guidelines for the main systematic review and it should be cited in the protocol as well.

 The data synthesis plan is not sufficiently explained in this protocol. For example, authors are planning to combine RCTs and observational studies in the meta-analysis that will add a huge clinical/methodological heterogeneity, and mean difference will be an appropriate measure. Similarly, what model will be used to combine data from different studies? All these important considerations must be incorporated with clarity in the protocol as a priory.

 GRADE approach better suits randomised control trials so authors must distinguish from observational studies and present a proper plan. Add reference for GRADE.

Is the study design appropriate for the research question?

Yes

Is the rationale for, and objectives of, the study clearly described?

Yes

Are sufficient details of the methods provided to allow replication by others?

No

Are the datasets clearly presented in a useable and accessible format?

Yes

Reviewer Expertise:

Epidemiology, Medical Statistics, Meta-analysis

I confirm that I have read this submission and believe that I have an appropriate level of expertise to confirm that it is of an acceptable scientific standard, however I have significant reservations, as outlined above.

# Response

**Published:** 2022-03-02

**Authors:** Kalpesh Panara

**Affiliations:** Institute of Teaching and Research in Ayurveda, India

First of all, we are gratefully appreciating your invaluable suggestions in improving the quality of our research article. The responses to your observation and suggestion are as follows:

**Comment #1: **Authors must be more specific about the study designs eligible for this systematic review. For example, it is completely unclear whether a large case-series study will be eligible or not. So, kindly elaborate on your inclusion in more detail.

**Response: **The authors are thankful for the observation of the reviewer. We wish to clarify that large case-series will also be excluded as it does not have the control arm.

 As far as the Explicit exclusion criteria of the articles are concerned, the following is our submission:

 “ *Protocol, pre-clinical, cross-sectional, case reports, case series, single-arm or not having appropriate control will be excluded. Articles published in a language other than English will also be excluded*.’’

 The intent of this reply is added and highlighted in the revised manuscript.

**Comment #2: **Authors cannot ignore Google Scholar for searching eligible articles, as it is one of the biggest databases for grey literature and lots of Indian papers published in non-indexed journals.

**Response: **In response to the reviewer’s suggestion, Google Scholar has been added in the source of information section, the relevant section has been highlighted in the revised manuscript.

**Comment #3:  **What do you mean by ‘till current time’ for searching different databases? There has to be an end date in the protocol.

**Response: **Here, we wish to clarify ‘till current time’ means the date on which the actual search will be done. However, considering the ambiguity of this phrase among the readers, we are deleting this in our revised article.

**Comment #4:**

 How was clinical improvement defined for this systematic review? Kindly explain in more detail.

**Response: **We are very thankful for the reviewer’s comment, and in response to the reviewer’s suggestion, the definition of clinical improvement has been added as follows:

 Clinical improvement has been defined as ‘achieving health status of an absence of symptoms attributed to COVID-19 and/or Saturation of Peripheral Oxygen (SpO _2_) > 93%’.

 The definition has been added and highlighted in the manuscript.

**Comment #5:  **The statement, “Protocol, pre-clinical, cross-sectional, case reports, case series, single-arm or not having appropriate control will be excluded. Articles published in a language other than English will also be excluded.” Should be mentioned in the eligibility section.

**Response: **The authors agree with the respected reviewer, and the statement mentioned above has been mentioned in the eligibility section.

**Comment #6: **Authors must adhere to the PRISMA 2020 reporting guidelines for the main systematic review and it should be cited in the protocol as well.

**Response: **Reporting guideline suggested by the reviewer has been added.

**Comment #7: **The data synthesis plan is not sufficiently explained in this protocol. For example, authors are planning to combine RCTs and observational studies in the meta-analysis that will add a huge clinical/methodological heterogeneity, and mean difference will be an appropriate measure. Similarly, what model will be used to combine data from different studies? All these important considerations must be incorporated with clarity in the protocol as a priory.

**Response: **The authors raised important concerns about the data synthesis method, and we have clarified it by adding more details on data syntheses.

 We agree that RCTs and NRSIs are not combined routinely in meta-analysis except in exceptional circumstances. As limited RCTs on Ayush interventions are anticipated, which may restrict us from performing a meta-analysis, we opted to add NRSI and RCT for meta-analysis. It may lead to induce clinical heterogeneity in the pooled outcome measure. Further, assuming heterogeneity due to different designs, we have planned a random effect model to compute the summary effect. Moreover, we will perform sensitivity analysis considering the study design to know the impact of the inclusion of NRSI on pooled estimates. Further, if sufficient RCTs will be available in subsequent updates, we may exclude NRSIs from data syntheses.

**Comment #8: **GRADE approach better suits randomized control trials so authors must distinguish from observational studies and present a proper plan. Add reference for GRADE.

**Response: **The authors are grateful to the reviewer for their observation.

 We will adopt the GRADE approach to know the quality and strength of evidence.

 The current GRADE approach for a body of evidence relating to interventions accept observational studies (otherwise known as non-randomized studies, or NRS) also wherein certainty for observational study starts with low grade, unlike RCTs. We will follow the GRADE present guideline for an observational study to rate the evidence.

 Reference for GRADE has been added.

   

 All the additional phases have been highlighted in green in the revised manuscript for easy review.

 Finally, the authors are warm-heartedly admiring your invaluable comments/suggestions and believe that your kind contributions have undisputedly helped us to improve the quality of the research article.

**Yours sincerely**

**Authors**

# Peer review: Reviewer response for version 1

**DOI:** 10.5256/f1000research.58655.r94658

**Published:** 2021-09-20

**Authors:** Rohit Sharma

**Affiliations:** 1 Department of Rasa Shastra and Bhaishajya Kalpana, Faculty of Ayurveda, Institute of Medical Sciences, Banaras Hindu University, Varanasi, Uttar Pradesh, India

The manuscript follows the PRISMA-P guidelines and is methodologically good and well written. The study covers two aims that encompass determining the Prophylactic effect of the AYUSH medicines on Sars-Cov-2 infection and evaluating the therapeutic effectiveness of AYUSH drugs to COVID-19 patients. As the study is living in nature, evidence will be updated from time to time and may guide stakeholders. However, there are a few concerns for authors to consider:

1. In the abstract author has written that 'Data will be synthesized, and the risk of bias will be assessed with RevMan 5.4 tool' that seems incomplete. RevMan tool has been designed for the synthesized and meta-analyzed data. The authors should mention the method used for the Risk of Bias assessment.
2. To give more impact on background, the author may include the COVID19 epidemics data of global and Indian tertiary.
3. In the introduction, there was no mention of chemo-prophylaxis or therapeutic treatments that are being used by conventional medicines that need to be added by authors.
4. The authors should provide additional information about the data synthesis methods used to analyse the various medicines in the AYUSH system, perpendicularly to whether it's evaluated together or separately?

I particularly value the fact that this is a good systematic review study protocol, possibly the first of its type to encompass the entire AYUSH system.

Is the study design appropriate for the research question?

Yes

Is the rationale for, and objectives of, the study clearly described?

Yes

Are sufficient details of the methods provided to allow replication by others?

Yes

Are the datasets clearly presented in a useable and accessible format?

Yes

Reviewer Expertise:

Ayurveda, Herbal Medicine, Traditional Medicine, Natural Products, Complementary and Alternative Medicine

I confirm that I have read this submission and believe that I have an appropriate level of expertise to confirm that it is of an acceptable scientific standard, however I have significant reservations, as outlined above.

# Response

**Published:** 2022-03-02

**Authors:** Kalpesh Panara

**Affiliations:** Institute of Teaching and Research in Ayurveda, India

First of all, we are gratefully appreciating your invaluable suggestions in improving the quality of our research article. The responses to your observation and suggestion are as follows:

**Comment #1: **In the abstract author has written that ‘Data will be synthesized, and the risk of bias will be assessed with RevMan 5.4 tool’ that seems incomplete. RevMan tool has been designed for the synthesized and meta-analyzed data. The authors should mention the method used for the Risk of Bias assessment.

**Response:  **As far as the methods of risk of bias assessment are concerned, the following is our submission:

 Revised Cochrane tool for assessing the risk of bias RoB-2 was used for RCTs, and Risk of Bias in Non-randomized Studies - of Interventions (ROBINS-I) was used for Non-Randomized Studies of Interventions (NRSIs) to rate studies at outcome level.

 The intent of this reply is added and highlighted in the abstract section of the revised manuscript.

**Comment #2: **To give more impact on background, the author may include the COVID-19 epidemics data of global and Indian tertiary.

**Response: **In response to the reviewer’s suggestion, we have added the following line that represents the current COVID-19 epidemics data of global and Indian territory in the introduction section. “Globally, more than 428 million cases had caused about 6 million COVID-19 deaths by end of the February 2022. In India, casualties crossed 5 lakh, out of 42 million confirmed.”

**Comment #3: **In the introduction, there was no mention of chemo-prophylaxis or therapeutic treatments that are being used by conventional medicines that need to be added by the authors.

**Response: **The authors are thankful for this observation and in response, chemo-prophylaxis or therapeutic treatments conventionally used have been added in the revised manuscript.

 “For inpatient situations, Remdesivir, Dexamethasone, and certain monoclonal antibodies are advocated; nevertheless, efficacy has been revealed to be limited. To expedite the discovery, hydroxychloroquine and azithromycin were repurposed to prevent or cure COVID-19, but the outcomes were not encouraging. Pre-exposure chemoprophylaxis therapies are still unavailable.”

**Comment #4:** The authors should provide additional information about the data synthesis methods used to analyse the various medicines in the AYUSH system, perpendicularly to whether it’s evaluated together or separately?

**Response: **In response, we have clarified the data synthesis methods used to analyze the various medicines in the AYUSH system in our revised manuscript. Following is our submission:

 We are intended to meta-analyse the outcome data of individual drugs when a sufficient number of studies reported the particular outcome. If a single study will report the particular outcome, we will only report the measure of effects. We anticipate scarce RCTs for each intervention, hence analytical observational studies will be included for data synthesis. As clinical heterogeneity is anticipated random effect model has been selected for meta-analysis.

 All the additional phases have been highlighted in green in the revised manuscript for easy review.

 Finally, the authors are warm-heartedly admiring your invaluable comments/suggestions and believe that your kind contributions have undisputedly helped us to improve the quality of the research article.

**Yours sincerely**

**Authors**
